# Supplementary material for: Kaempferol and Its Glycoside, Kaempferol 7-O-rhamnoside, Inhibit PD-1/PD-L1 Interaction In Vitro
Source: Int J Mol Sci. 2020 May 3;21(9):3239. doi: 10.3390/ijms21093239 (PMC7247329; doi:10.3390/ijms21093239)
Supplement: Supplementary file 1 [file ijms-21-03239-s001.pdf]

## S.1. Supplementary data

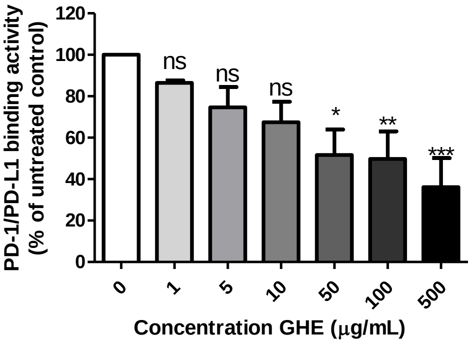


**Figure S1.** Effects of GHE on PD-1/PD-L1 protein interaction in a competitive ELISA. GHE were pre-treated onto plates coated with PD-L1, followed by incubation with biotinylated PD-1. Relative PD-1/PD-L1 binding activities were determined using a competitive ELISA assay, as described in the Materials and Methods. Data are presented as means ± S.E. (standard error) values of three independent experiments. Asterisks indicate significant inhibition of PD-1/PD-L1 binding activity by each test inhibitor as compared with the untreated control group. (*$p < 0.05$, **$p < 0.01$, and ***$p < 0.001$).

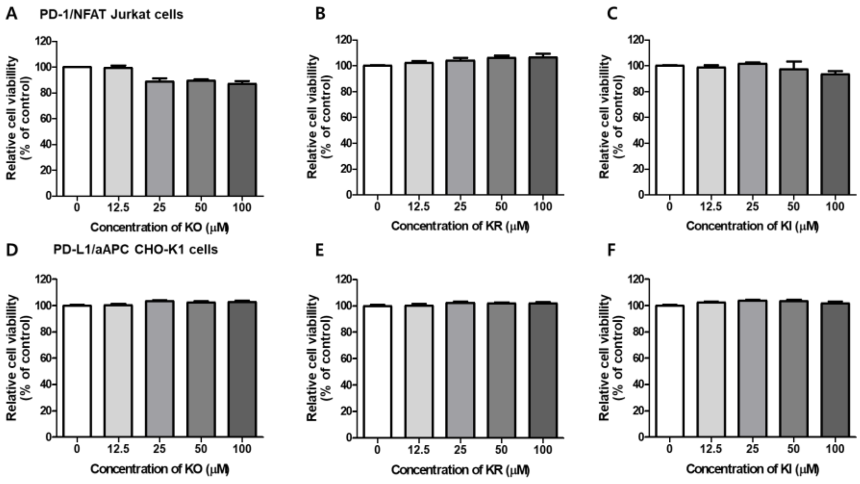


**Figure S2.** Effects of KO, KR, and KI on cell viability in PD-1 Jurkat cells and PD-L1/aAPC CHO-K1 cells.
(A) PD-1 Jurkat effector cells and (B) PD-L1/aAPC CHO-K1 target cells were treated with each compound for 24 hours at the indicated concentrations. Cell viability was measured by the CCK assay, as explained in the Materials and Methods. Data are presented as means ± S.E. (standard error) values of three independent experiments. All group did not show the significant inhibition of cell viability by each samples as compared with the control group.

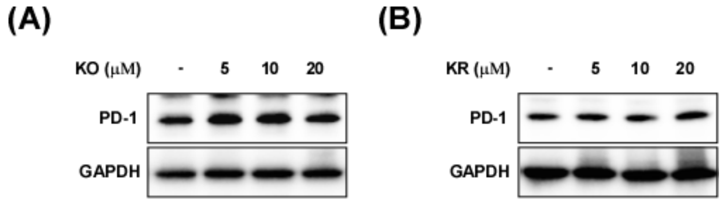


**Figure S3.** Effect of KO or KR on PD-1 expression in co-cultured PD-1 Jurkat cells and PD-L1 CHO-K1 cells. Co-cultured cells were treated with KO or KR at the indicated concentrations for 24 h. Cell lysates were determined by Western blot analysis as described in Supplementary Materials.

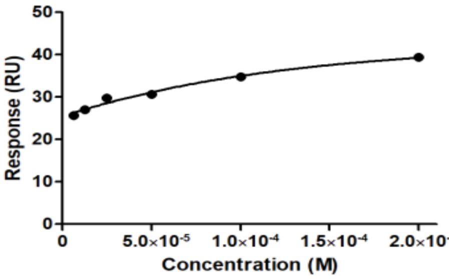


**Figure S4.** The binding of KO or KR against human PD-1 analyzed by SPR. The binding of KR with Fc-PD-1 was evaluated using SPR assay. KR were allowed to flow over Fc-PD-L1 captured on a flow cell as well as on a reference cell of Series S Sensor Chip Protein A at indicated concentrations, and the Rmax (24.92) and KD values (156 μM) were determined by Biacore software.

## S.2. Supplementary Table

**Table S1.** Estimated binding energy and interactions between amino acids of PD-1 protein and each compounds.

| Ligand | Target proteins score (kcal/mol) | Amino acid residues | |
|---|---|---|---|
| | | Hydrophobic | Hydrogen bond |
| Kaempaferol (KO) | -5.4 | Tyr68, Gly124, Ile126, Leu128, Ile134 | Thr76, Glu136 |
| Kaempferol 7-O-rhamnoside (KR) | -5.6 | Val64, Gly124, Ile126, Leu128, Ile134 | Tyr68, Thr76, Glu136 |
| PD-1/PD-L1 Inhibitor C1 | -6.0 | | |

n/a[1]; not applicable

**Table S2.** Estimated binding energy and interactions between amino acids of PD-L1 protein and each compounds.

| Ligand | Target proteins score (kcal/mol) | Amino acid residues | |
|---|---|---|---|
| | | Hydrophobic | Hydrogen bond |
| Kaempaferol (KO) | -5.0 | Tyr56, Arg113, Met115, Tyr123 | Gln66 |
| Kaempferol 7-O-rhamnoside (KR) | -5.3 | Ile54, Tyr56, Gln66, Glu58, Arg113, Met115, Ala121, Tyr123 | n/a[1] |
| PD-1/PD-L1 Inhibitor C1 | -6.0 | | |

n/a[1]; not applicable

## S.3. Supplementary Materials

### *S.3.1. Cell viability assay*

Cell viability was measured using Cell Counting Kit-8 (CCK) according to the supplier's instructions (Dojindo Molecular Technologies, Inc., Rockville, MD, USA) [S1]. CCK is one of sensitive colorimetric assay for the determination of cell viability. WST-8, highly water-soluble tetrazolium salt, is converted by dehydrogenase activities in cells to a yellow-color formazan dye. Briefly, cells were seeded into 96-well plates at a density of $1 \times 10^4$ cells/well and cultured overnight before GHE treatment. Test inhibitors were added to the wells at the indicated concentrations. After incubation for the indicated time, 10 μL of CCK solution was treated for 2 h at 37°C. The amount of the formazan dye,

generated by the activities of dehydrogenases in cells, is soluble in the cell culture media and it is directly proportional to the number of living cells. To measure the color density at 450 nm, a microplate reader from Molecular Devices i3 (San Jose, California, USA) was used.

*S.3.2. Surface Plasmon Resonance (SPR) analysis*

The binding affinities between each compound and hPD-1 (Biomatik #RPU43427) or hPD-L1 (Biomatik #RPU53816) were assayed using a Biacore T200 biosensor equipment from GE Healthcare (Chicago, Illinois, U.S.). The purified hPD-1 was diluted in dilution buffer (20 mM HEPES pH 8.0, 150 mM NaCl) to the final dose of 25 μg/mL. The diluted proteins was immobilized on a CM5 sensor chip by amino coupling reagent kit, and the immobilization level was 5000 response unit (RU). Experiments for binding were performed in HBS-EP buffer (0.01 M HEPES pH 7.4, 0.15 M NaCl, 3 mM EDTA, 0.005% v/v Surfactant P20) at 25°C with a flow rate of 30 μl/min. To determine the binding affinities, gradient concentrations of each compounds (0.5 μM~200 μM) were injected into the channel for 100s, followed by disassociation for 300s. RU values were measured and all the experimental results were globally analyzed by program of Biacore T200 Evaluation software, version 2.0 [S2].

*S.3.3. Western blot analysis*

Co-cultured cells ($5 \times 10^4$ cell/mL) were seeded in a 6 well plate and then treated with KO or KR for 24 h at the indicated concentration (0, 5, 10, 20 μM). Protein preparation and Western blot were performed as previously reported [S3]. The cells were scraped in a lysis buffer (10 mM Tris [pH 7.5], 150 mM NaCl, 5 mM ethylene diamine tetra acetic acid, 1% Triton X-100, 1 mM dithiothreitol, 0.1 mM phenylmethylsulfonyl fluoride, 10% glycerol, and protease inhibitor cocktail tablet), incubated on ice for 20 minutes, and then centrifuged at 480 xg for 20 min. The protein concentrations were measured using a dye-binding protein assay kit purchased from Bio-Rad Laboratories (Hercules, CA) as described by the manufacturer. The proteins were separated by electrophoresis in a 10% sodium dodecyl sulfate-polyacrylamide gel and transferred to a polyvinylidene fluoride (PVDF) membrane from Millipore (Billerica, MA). The membrane was blocked with 5% skim milk for 1 h 30 min and then incubated with the specific primary antibody at 4°C overnight. After hybridization with the secondary antibody, protein bands were visualized using a chemiluminescence detection kit from Bio-Rad (Hercules, CA, USA). Primary antibody including anti-PD-1 and anti-GAPDH (diluted 1:1000) were purchased from Cell Signaling Technology (Danvers, MA, USA). Secondary antibody for anti-rabbit and anti-mouse (diluted 1:5000) were purchased from GenDEPOT Inc. (Katy, TX, USA).

## S.4. Supplementray Reference


1. Choi, J.G.; Kim, Y.S.; Kim, J.H.; Chung, H.S. Antiviral activity of ethanol extract of Geranii Herba and its components against influenza viruses via neuraminidase inhibition. *Sci. Rep.* **2019**, *9*, 12132.
2. Li, Q.; Quan, L.; Lyu, J.; He, Z.; Wang, X.; Meng, J.; Zhao, Z.; Zhu, L.; Liu, X.; Li, H. Discovery of peptide inhibitors targeting human programmed death 1 (PD-1) receptor. *Oncotarget* **2016**, *7*, 64967–64976.
3. Kim, J.H.; Kim, D.H.; Cho, K.M.; Kim, K.H.; Kang, N.J. Effect of 3,6-anhydro-l-galactose on $\alpha$-melanocyte stimulating hormone-induced melanogenesis in human melanocytes and a skin-equivalent model. *J. Cell Biochem.* **2018**, *119*, 7643–7656.
